# ASPIRER: a new computational approach for identifying non-classical secreted proteins based on deep learning

**DOI:** 10.1093/bib/bbac031

**Published:** 2022-02-17

**Authors:** Xiaoyu Wang, Fuyi Li, Jing Xu, Jia Rong, Geoffrey I Webb, Zongyuan Ge, Jian Li, Jiangning Song

**Affiliations:** Monash Biomedicine Discovery Institute and Department of Biochemistry and Molecular Biology, Monash University, Melbourne, VIC 3800, Australia; Department of Microbiology and Immunology, The Peter Doherty Institute for Infection and Immunity, The University of Melbourne, Melbourne, Victoria, Australia; Monash Biomedicine Discovery Institute and Department of Biochemistry and Molecular Biology, Monash University, Melbourne, VIC 3800, Australia; Department of Data Science and AI, Faculty of Information Technology, Monash University, Melbourne, VIC 3800, Australia; Department of Data Science and AI, Faculty of Information Technology, Monash University, Melbourne, VIC 3800, Australia; Monash e-Research Centre and Faculty of Engineering, Monash University, Melbourne, VIC 3800, Australia; Biomedicine Discovery Institute and Department of Microbiology, Monash University, Melbourne, VIC 3800, Australia; Department of Data Science and AI, Faculty of Information Technology, Monash University, Melbourne, VIC 3800, Australia

**Keywords:** non-classical secreted protein, bioinformatics, machine learning, deep learning, feature engineering, predictor

## Abstract

Protein secretion has a pivotal role in many biological processes and is particularly important for intercellular communication, from the cytoplasm to the host or external environment. Gram-positive bacteria can secrete proteins through multiple secretion pathways. The non-classical secretion pathway has recently received increasing attention among these secretion pathways, but its exact mechanism remains unclear. Non-classical secreted proteins (NCSPs) are a class of secreted proteins lacking signal peptides and motifs. Several NCSP predictors have been proposed to identify NCSPs and most of them employed the whole amino acid sequence of NCSPs to construct the model. However, the sequence length of different proteins varies greatly. In addition, not all regions of the protein are equally important and some local regions are not relevant to the secretion. The functional regions of the protein, particularly in the N- and C-terminal regions, contain important determinants for secretion. In this study, we propose a new hybrid deep learning-based framework, referred to as ASPIRER, which improves the prediction of NCSPs from amino acid sequences. More specifically, it combines a whole sequence-based XGBoost model and an N-terminal sequence-based convolutional neural network model; 5-fold cross-validation and independent tests demonstrate that ASPIRER achieves superior performance than existing state-of-the-art approaches. The source code and curated datasets of ASPIRER are publicly available at https://github.com/yanwu20/ASPIRER/. ASPIRER is anticipated to be a useful tool for improved prediction of novel putative NCSPs from sequences information and prioritization of candidate proteins for follow-up experimental validation.

## Introduction

Bacteria can be classified as Gram-positive or Gram-negative according to the properties of the peptidoglycan layer using the Gram staining [1], which is a common technique for phenotypic characterization of bacteria. Generally, the peptidoglycan layer of Gram-positive bacteria is thicker than that of Gram-negative bacteria, while the outer membrane is absent compared with Gram-negative bacteria [2]. Because of fast growth rate and genetic modifiability, bacteria are commonly used as a cell factory to produce heterogeneous proteins based on secretion systems [3]. Due to the lack of the outer membrane, Gram-positive bacteria are considered as desirable microbial hosts in industrial biotechnology [4].

Previous studies have shown that bacteria can export proteins via two major secretion pathways, namely the general secretion (Sec) pathway [5] and the twin-arginine translocation (Tat) pathway [6]. The Sec-dependent pathway catalyzes the transformation of the unfolded protein, which folds in the trans-side of the membrane. In contrast, the Tat-dependent pathway is responsible for exporting the folded proteins across the cytoplasmic membrane. A commonality of these proteins is that they both have signal peptides consisting of n-region, h-region and c-region [7]. The proteins secreted by the known secretion pathways with signal peptides or secreted motifs are termed classical secreted proteins (CSPs). In contrast, cytoplasmic proteins are identified in supernatant without any signal peptide and secretion motif and as termed non-CSP (NCSPs). Several previous studies have shown that the secretion of these cytoplasmic proteins is not simply attributed to cell lysis [3, 8, 9]. Furthermore, a number of hypotheses have been proposed for the secretory pathway and the recognition of non-classical proteins [3]; however, different from the classical secretion pathway, to date, the mechanism of the non-classical secretion pathway remains largely unknown.

The first NCSP, namely bacterial glyceraldehyde-3-phosphate dehydrogenase (GAPDH), was discovered by Pancholi and Fischetti in 1992 [10]. Antelmann *et al*. [11] experimentally identified 17 cytoplasmic proteins, which had no signal peptides in *Bacillus subtilis*. More recently, Wang *et al*. [12] summarized 45 common NCSPs identified from three different bacterial species. Due to the increasing demand for high-level secretion of recombinant proteins, bacteria have been extensively used to produce such proteins as an effective tool. The secretion strategy dependent on signal peptide is not straightforward, with each step of the classical secretion pathway relying on dozens of translocation components, resulting in low yields of the protein [13]. Due to the bottleneck in the Sec- and Tat-dependent recombination protein expression systems, researchers are shifting their priorities to use non-classical secretion system to assist the secretion of the proteins of interest. Compared with time-consuming, expensive and sophisticated experimental approaches, computational methods require less processing time and lower cost, and as such, can enable genome-wide identification of NCSPs in a high-throughput and cost-effective manner.

Bendtsen *et al*. [14] proposed the first computational method, termed SecretomeP, to identify mammalian secretory proteins using sequenced-based features. The training dataset of SecretomeP was curated based on the subcellular localization annotation as only a limited number of NCSPs were characterized at that time, and the corresponding signal peptide parts of those mammalian extracellular proteins were removed. SecretP [15] is a support vector machine (SVM)-based approach developed to distinguish the NCSPs, CSPs and non-secreted proteins by taking into account both sequence and structural properties. A brief summary of the existing computational methods for the NCSPs is provided in Table 1 with respect to several main aspects, including the training and test datasets, the features used for model training, the algorithms and the webserver and software availability.

**Table 1:** A comprehensive list of the predictors for the prediction of NCSPs in the literature

Tool	Training set	Testing set	Features	Method	Evaluation strategy	Software availability	Webserver availability	Year
SecretomeP [14]	3321 Extracellular mammalian proteins and 3654 cytoplasmic/nuclear mammalian proteins	13 Non-classical human secretory proteins	Number of atoms, number of postive residues, low-complexity regions, transmembrane helices, protein sorting, propeptide cleavage site	Neural network	Cross-validation and independent test	Commerical	Yes	2004
SecretomeP 2.0 [16]	152 Extracellular proteins and 140 cytoplasmic proteins from *Firmicutes*; 350 extracellular proteins and 334 cytoplasmic proteins from *Proteobacteria*	35 Non-classical secretory proteins in Gram-positive bacteria and Gram-negative bactreria	Gram-positive bacteria: threonine content, composition, transmembrane helices, grand average of hydropathy (Gravy), protein disorder, secondary structure Gram-negative bacteria: arginine content, composition, instability index, protein disorder	Artificial Neural network	3-Fold cross-validation and independent test	No	Yes	2005
SecretP [15]	230 Mammalian secreted proteins without signal peptides and 685 extracellular proteins with signal peptides	92 Human secreted proteins without signal peptide	Pse-AAC	SVM	5-Fold cross-validation and independent test	No	Not available now	2010
NClassG+ [17]	420 Secreted proteins and 433 cytoplasmic proteins of Gram-positive bacteria	82 Secreted proteins without signal sequence and 263 cytoplasmic proteins	AAC, dipeptide, physicochemincal features and PSSM	SVM	Nested *k*-fold cross-validation and independent test	No	Not available now	2011
PeNGaRoo [18]	141 NCSPs and 446 cytoplasmic proteins of Gram-positive bacteria	34 NCSPs and 34 cytoplasmic proteins of Gram-positive bacteria	PAAC, QSO, TPC, Pse-PSSM, AATP, CTriad, CTDT	A two-layer lightGBM model	10-Fold cross-validation, leave-one-out cross-validation and independent test	No	Yes	2020
NonClasGP-Pred [19]	Same as PeNGaRoo	Same as PeNGaRoo	ACC, DPC, CTDC, CTDD, CTriad, PAAC, CKSAAP, NMBroto, QsOrder	SVM	10-Fold cross-validation and independent test	No	Yes	2020

Based on SecretomeP, Bendtsen *et al*. [16] developed SecretomeP 2.0, which expanded the repertoire of the predicted NCSPs in both Gram-positive and Gram-negative bacteria. In another study, Montoya *et al*. [17] developed a sequence-based classifier, called NClassG+, which can predict NCSPs in Gram-positive bacteria. Benefiting from the development of experimental technologies for characterizing NCSPs, an increasing number of NCSPs have been recently identified. This provides an excellent opportunity to develop more accurate prediction models to accelerate the discovery of new NCSPs. Moreover, more recent attention has focused on the NCSPs in bacteria, especially in Gram-positive bacteria. Based on the study of Wang *et al*. [12], Zhang *et al*. [18] implemented a combined gradient boost and ensemble learning framework, called PeNGaRoo, to predict the NCSPs in Gram-positive bacteria. More recently, another NCSPs predictor termed NonClasGP-pred [19] has been developed based on the integration of subset-specific optimal SVM models.

To date, several challenging problems remain to be addressed. For example, the performance of current NCSP predictors was relatively low on the independent test. Another important issue is that most NCSP predictors utilized the whole amino acid sequence to extract the features and train the models of NCSP prediction. However, the length of NCSPs can vary substantially from tens to thousands of amino acid residues, and it is likely that certain local regions might benefit the secretion of NCSPs. For instance, previous studies have reported that the N- and C-terminal residues are crucial for secretion, while deletion of N- and C-terminal residues results in the inhibition of secretion [8]. Therefore, features extracted from terminal sequences may be useful for improving the prediction of NCSPs.

In the present study, we propose a novel hybrid deep learning-based predictor, termed ASPIRER, for the improved prediction of NCSPs. Specifically, ASPIRER combines a whole amino acid sequence-based Extreme Gradient Boosting (XGBoost) model with an N-terminal sequence-based convolutional neural network (CNN) model. For the XGBoost model, a variety of informative feature descriptors are extracted from the whole amino acid sequence to characterize the NCSPs and train the model. Moreover, feature selection and Synthetic Minority Over-sampling Technique (SMOTE) [20] algorithms are applied. For the N-terminal sequence-based CNN model, 60 N-terminal residues of NCSPs are extracted and used as the input to train the CNN model. In addition, the random oversampling technique is implemented for the CNN model. For the XGBoost model, the handcrafted features are used to represent the properties of NCSPs based on the whole amino acid sequence. In contrast, the N-terminal sequence-based CNN model can recognize specific patterns from the N-terminal sequence. Benchmarking experiments indicate that this hybrid deep learning-based model outperforms existing state-of-the-art models and commonly used sequence alignment methods.

## Materials and methods

**Figure 1:**
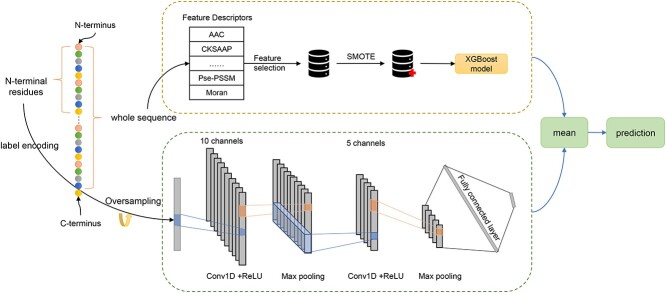
An overall framework of ASPIRER.

An overall framework of the ASPIRER methodology is illustrated in Figure 1. As can be seen, ASPIRER comprises two sub-models —a whole sequence-based XGBoost model and an N-terminal local sequence-based CNN model. The two sub-models, respectively, take the whole sequence and N-terminal sequence as the input. The final output is generated by integrating the outputs of the two sub-models.

### Data collection and processing

In this study, the dataset was collected from the study of Zhang *et al*. [18]. All NCSPs in the dataset were initially collected from the UniProt database (UniProt, 2015), identified in at least three bacterial species and three research groups [12]. The negative samples were obtained from Bendtsen *et al*. [16] (cytoplasmic proteins in *Firmicutes*). After removing the sequence redundancy in the resulting dataset using the CD-HIT program [21], 157 positive and 446 negative non-redundant samples were obtained. Among these, 141 validated NCSPs and 446 cytoplasmic proteins were used as the training dataset, similar to the previous work [18]. In addition, the positive samples of independent test dataset were constructed by randomly selecting one-tenth of the NCSPs and experimentally validated NCSPs from previous studies and Zhang *et al.*’s work. In contrast, the negative samples were collected from UniProt, which were annotated as ‘cytoplasm’ or ‘cytoplasmic’ but not annotated as ‘secreted’. The independent test dataset was the same as Zhang *et al.*’s work [18]. The sequence lengths of the positive and negative samples had similar distributions to avoid potential bias. The corresponding protein IDs and amino acid sequences can be downloaded from GitHub at https://github.com/yanwu20/ASPIRER/.

### Feature engineering

We used two different feature extraction strategies to encode the protein sequences, including the handcrafted features for training the XGBoost model and sequence-to-vector encoding for training the CNN model. The handcrafted features used in the XGBoost model included 13 types of features which could be categorized into five major groups—amino acid composition (AAC), physicochemical property, evolution-based, grouped AAC and autocorrelation features. AAC, physicochemical property and evolution-based features have been widely used in previous studies and demonstrated their utility for NCSP prediction [17–19]. Since some amino acid residues have similar physicochemical properties, the change between amino acid residues of the same group might have less impact on their functions. Therefore, the grouped AAC features can reflect the protein’s properties better in some situations. The autocorrelation feature descriptors describe the difference of amino acid sequences based on their physicochemical properties and specific structure. They have been applied in numerous previous studies, such as predicting specific protein family, oligomeric states of proteins [22, 23] and protein–protein interactions [24].

AAC features reflect the frequency of amino acid types and pairs. In this study, we calculated the AAC, the Composition of K-Spaced Amino Acid Pairs (CKSAAP), Di-Peptide Composition (DPC), Tri-Peptide composition (TPC) and Dipeptide Deviation from Expected Mean. For the physicochemical property features, the Composition (CTDC), Transition (CTDT), Distribution (CTDD) and Conjoint Triad (CTriad) were selected, and these features can represent the distribution patterns and physicochemical properties of the amino acids. The evolution-based feature is Pseudo Position-Specific Score Matrix (Pse-PSSM) [25], which describes the evolution and sequence-order information. Similar to AAC features, the group AAC features are frequencies of amino acid types. The amino acids are categorized into several groups based on their physicochemical properties. The Grouped Di-Peptide Composition belongs to the group AAC. The fifth feature group is autocorrelation, and, in this group, the Moran correlation descriptor is adopted, which is based on the distribution of amino acid properties. The majority of the feature descriptors used in this study can be calculated using feature engineering/machine learning tools iFeature [26], iLearn [27] and iLearnPlus [28], with the only exception of the Pse-PSSM features, which were extracted using the POSSUM software package [29]. A detailed description of each of the feature descriptors used is provided in the Supplementary Material.

The handcrafted features are not commonly used for training the N-terminal sequence-based CNN model, especially when the curated dataset is limited. In this case, the automatically learned feature by the deep learning model might be better than the handcrafted features. Thus, the amino acids were directly transformed into 21 vectors corresponding to 20 kinds of amino acids plus one ambiguous amino acid and then fed into the CNN model.

### The architecture of the ASPIRER

The hybrid framework of ASPIRER comprises two parts, including the whole amino acid sequence-based XGBoost model and an N-terminal sequence-based CNN-based model. The outputs of these two sub-models are combined using the averaging scheme. The benefit of this approach is that both important properties of the whole amino acid sequence and determinants in the N-terminal region of the protein are taken into consideration for NCSPs prediction. The two sub-models are briefly introduced in the following two sections, while performance assessment is detailed in the Supplementary material.

### XGBoost-based whole amino acid sequence model

Extreme gradient boosting (XGBoost) is a gradient boosted tree algorithm that has been widely used for solving classification problems [30, 31]. As high-dimensional input variables can increase the computational cost and affect the model performance, a feature selection strategy was applied to reduce the dimension of the input variables and remove the redundant features. For this XGBoost model, the chi-square (Chi2) test [32] was adopted for feature selection, and the number of selected features was determined by the cross-validated grid search. The Chi2 feature selection method calculates the dependence between the features and the label and is formulated as follows:

$$ {\chi}^2=\sum_{i=1}^n\frac{{\left({\mathrm{obs}}_i-{\exp}_i\right)}^2}{\exp_i}, $$

where ${\mathrm{obs}}_i$ denotes the observed frequency of the sample *i*, ${\exp}_i$ is the expected frequency of the sample *i* and *n* is the number of the samples. The features were ranked by the dependence score, and the top *k* features with the highest values were selected.

The SMOTE strategy was further adopted to balance the dataset to address the data imbalance problem by oversampling the positive samples. Grid search was performed to optimize the hyperparameters based on the 5-fold cross-validation, and in this process, the AUROC was employed as the primary measure to determine the optimal hyperparameters, which included the maximum depth of each tree, subsampling, rate and minimum child weight.

### N-terminal sequence-based CNN model

As a powerful deep learning technique, CNN has been widely applied in computer vision and has also been successfully employed to address sequence-based bioinformatics problems, such as the protein/DNA/RNA functional site prediction [33–35], protein binding sites prediction [34, 36], protein structure prediction [37, 38] and promoter identification [39].

For the N-terminal sequence-based CNN model, we applied the random oversampling method to balance the dataset. We generated the new positive samples based on the random sampling from the positive set to ensure that the same numbers of positive and negative samples were obtained. As we were interested in extracting the specific pattern of the N-terminal sequence, the oversampling method should not change the pattern of the sequence. From this perspective, the random oversampling method is more suitable than the SMOTE strategy, which can potentially alter the sequence pattern of the N-terminal sequence.

For the CNN-based N-terminal sequence model, the first layer is an embedding layer that aims to transform the input into dense vectors of fixed size (i.e. 64 dimensions). After that, the architecture consists of two one-dimensional convolutional layers (Conv1D), two max-pooling layers and a fully connected layer. We used the rectified linear units (ReLU) as the active function for the two Conv1D layers. The kernel size of the Conv1D layer is 16; there were 10 and 5 filters for the two Conv1D layers, respectively. The Adam algorithm was adopted for the hyperparameter optimization [34, 35, 40]. The hyperparameter tuning was performed based on the 5-fold cross-validation. The optimal learning rate was 0.001, and the batch size was 32. The early stop strategy was implemented if the validation loss stopped decreasing in two consecutive epochs. The CNN model was implemented based on the Keras library in Python [41].

## Results and discussion

In this section, we elaborate on the design rationale of ASPIRER and discuss the results for the identification of NCSPs.

### Performance of the XGBoost-based whole sequence model

**Figure 2:**
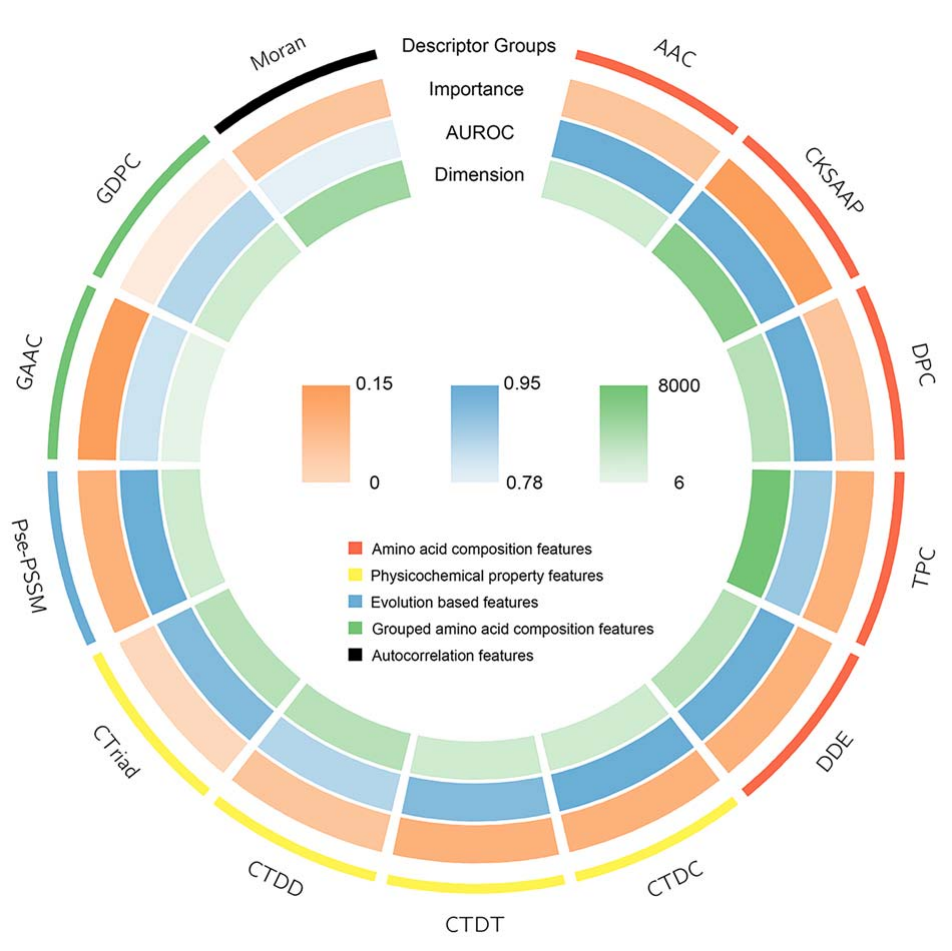
Comparison of the feature descriptors in terms of feature importance, performance of single descriptor model and feature dimension.

Several effective feature encodings were applied to extract informative features from the protein sequences to construct a reliable model. Suppplementary Table S2 (see Supplementary Data available online at http://bib.oxfordjournals.org/) shows the performance of the models trained using single types of feature descriptors and the whole amino acid sequence-based model on 5-fold cross-validation. The XGBoost model with all features but without feature selection is also included. As shown in Suppplementary Table S2 (see Supplementary Data available online at http://bib.oxfordjournals.org/), all the single descriptor-based models achieved a relatively good performance. The AUROC was higher than 0.8, which indicates that these descriptors could extract useful information to identify NCSPs. The XGBoost model trained using all features achieved a better performance than models trained using single feature descriptors with an AUROC of 0.937. To further evaluate the contribution of each feature type, we compared the AUROC results of 5-fold cross-validation, descriptor importance and feature dimensions. We quantified the descriptor importance by eliminating this descriptor from the XGBoost model with all descriptors and calculating the difference of the AUROC values. From Figure 2, we can see that all descriptor importance is positive, which means that all descriptors can improve the model performance. Therefore, we retained all the feature descriptors to train the final XGBoost model.

**Figure 3:**
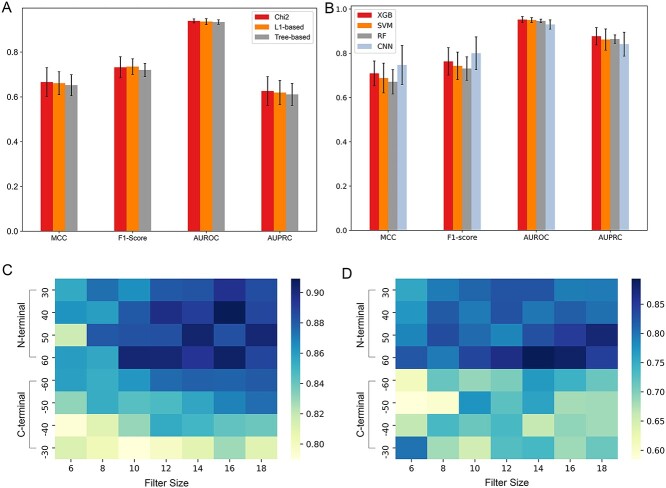
(A) Performance comparison of different feature selection strategies based on XGBoost on 5-fold cross-validation; (B) performance comparison of the final hybrid model with different whole sequence models on 5-fold cross-validation; (C) performance comparison of the CNN models based on different N- and C-terminal sequences and filter sizes on 5-fold cross-validation and (D) performance comparison of such models on the independent test.

We adopted different feature selection methods to improve the model performance, including Chi-square (Chi2), L1-based feature selection and Tree-based feature selection. The Chi2 feature selection is a univariate feature selection that selects the top *k* highest values based on Chi-square statistics. The L1-based feature selection method eliminates the feature with zero coefficient based on the Liner model penalized with the L1 normalization. The tree-based feature selection can calculate impurity-based importance and discard irrelevant features. The feature selection methods were implemented based on the Scikit-learn package. The results on the 5-fold cross-validation are illustrated in Figure 3A. As can be seen, the model trained using the selected features by the Chi2 feature selection method achieved the best performance in terms of the Matthew’s correlation coefficient (MCC), AUROC and AUPRC. In addition, it also achieved the best performance in terms of AUROC on the independent test (Suppplementary Table S4, see Supplementary Data available online at http://bib.oxfordjournals.org/), indicating that the Chi2 feature selection is the best feature selection method in this study.

We further assessed the performance of the XGBoost model by combining all handcrafted features and the XGBoost model by integrating the features selected based on the Chi2 feature selection strategy (Suppplementary Table S5, see Supplementary Data available online at http://bib.oxfordjournals.org/). As a result, we found that the model trained using the selected features with the feature selection method achieved a slightly improved performance, with an AUROC of 0.939. To enhance the performance XGBoost model, the SMOTE algorithm was also adopted to balance the dataset. The performance results on the 5-fold cross-validation and independent tests are provided in Supplementary Tables S5 and S6 (see Supplementary Data available online at http://bib.oxfordjournals.org/), respectively. To summarize, the results show that the model based on feature selection and SMOTE algorithm achieved the best performance in terms of AUROC on the cross-validation and independent tests. To evaluate the importance of feature selection and SMOTE algorithm, we compared the performance of these two models with the final model. The performance comparison results are provided in Suppplementary Table S7 (see Supplementary Data available online at http://bib.oxfordjournals.org/). We can see that the model with feature selection and SMOTE (‘FS + SMOTE’) achieved the best performance compared with the other two models except for specificity and precision. Supplementary Figure S1 (see Supplementary Data available online at http://bib.oxfordjournals.org/) shows the feature distributions after the feature selection and SMOTE algorithm based on the *t*-distributed stochastic neighbour embedding (T-SNE) algorithm [42]. The results also show that the feature selection and SMOTE algorithm contributed to the prediction of NCSPs.

### Performance evaluation of different machine learning methods

Previous works have shown that machine learning algorithms with handcrafted features achieved good performance for predicting the NCSPs from the entire sequence [18, 19]. Herein, we also constructed the whole amino acid sequence-based model and examined the impact of the properties at the whole-sequence level on the prediction of NCSPs.

We implemented several popular machine learning models using the same dataset and data processing strategy to ascertain the best-performing machine learning algorithm at the whole amino acid sequence level. Specifically, the XGBoost model was compared with the other two machine learning models trained using Random Forest (RF) and SVM. The RF algorithm proposed by Breiman *et al*. [43] is an ensemble classifier that constructs multiple decision trees using the bagging strategy. It has been widely used in protein sequence analysis, such as protein–protein interaction prediction [44, 45], disease protein identification [46–48], antimicrobial peptide and protein post-translational modification prediction [49–51]. SVM is a supervised learning algorithm that aims to find the optimal hyperplane to separate the positive and negative data points [52]. The parameters of all these compared machine learning models were optimized based on the 5-fold cross-validated grid search. The SMOTE and feature selection methods were also applied to the RF and SVM models. To better compare the performance of these different algorithms, we also included the CNN model trained using the whole amino acid sequence.

The performance results of different whole sequence-based models trained using different machine learning algorithms on 5-fold cross-validation and independent test are shown in Supplementary Tables S8 and S9 (see Supplementary Data available online at http://bib.oxfordjournals.org/), respectively. There was not much performance difference among the CNN, XGB and SVM models on 5-fold cross-validation. Thus, to better evaluate the influence of machine learning algorithms on the performance of the whole sequence model, we further compared the performance of the final hybrid model with different whole sequence models on 5-fold cross-validation. The results are shown in Figure 3B and Suppplementary Table S10 (see Supplementary Data available online at http://bib.oxfordjournals.org/). The final hybrid model with the XGBoost-based whole sequence model exhibited a stable and superior performance compared with the other models. The XGBoost model also outperformed the other three models on the independent test, with an AUROC of 0.9066, recall of 0.6471 in, MCC of 0.6155, accuracy of 0.7941, F1-score of 0.7586 and AUPRC of 0.9157, respectively (Suppplementary Table S6, see Supplementary Data available online at http://bib.oxfordjournals.org/). These results indicate that the XGBoost model can provide more robust performance for predicting NCSPs at the whole sequence level.

### Performance of the N-terminal sequence-based CNN model

Considering that the N-terminal residues are essential, we developed an N-terminal sequence-based CNN model. The length of the N-terminal sequence and size of the convolution filter are two critical parameters that influence the CNN model performance. The performance results of CNN models with different sequence lengths and filter sizes on the 5-fold cross-validation and independent tests are illustrated in Figure 3C and D, respectively. The results show that the models based on 40 and 60 N-terminal residues led to superior performance than the other settings. As shown in Suppplementary Table S11 (see Supplementary Data available online at http://bib.oxfordjournals.org/), when using the filter size of 16, the models based on 40 and 60 N-terminal residues achieved a similar performance; however, the performance of the model based on 60 N-terminal residues appeared to be slightly better than that of the model based on 40 N-terminal residues in terms of AUROC (Supplementary Figure S3, see Supplementary Data available online at http://bib.oxfordjournals.org/). Therefore, we used the window size of 60 to construct the final N-terminal sequence-based CNN model. Together, the results indicate that the 60 N-terminal residues can indeed provide useful information for the prediction of NCSPs. In addition, the results also suggest that the N-terminal sequence-based model is more suitable for the NCSP prediction than the C-terminal sequence-based model. This is also consistent with the sequence logo result (Supplementary Figure S2, see Supplementary Data available online at http://bib.oxfordjournals.org/).

To investigate whether the performance of CNN model could be further improved using the oversampling method, we compared the performance of the N-terminal sequence-based models with and without the random oversampling method. The results are provided in Suppplementary Table S12 (see Supplementary Data available online at http://bib.oxfordjournals.org/). We can see that the N-terminal sequence-based model with random oversampling achieved the best performance in terms of Recall, AUROC and AUPRC. For the final hybrid model, random oversampling also improved the performance, which achieved a superior performance than the model without random oversampling (Suppplementary Table S13, see Supplementary Data available online at http://bib.oxfordjournals.org/).

**Figure 4:**
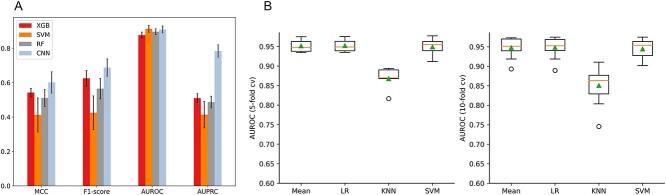
(A) Performance comparison of different N-terminal model on 5-fold cross-validation and (B) performance comparison of different ensemble strategies on 5- and 10-fold cross-validation.

To further examine the effectiveness of the CNN model for improving the performance of the N-terminal sequence model, we also trained machine learning models with handcrafted features extracted from the 60 N-terminal residues (Figure 4A). The results show that the CNN model achieved superior performance in terms of MCC, F1-score and AUPRC on the 5-fold cross-validation. Moreover, the CNN model trained using the 60 N-terminal residues clearly outperformed all other models in terms of six different performance metrics on the independent test with a Recall of 0.6176, Accuracy of 0.7941, MCC of 0.6287, F1-score of 0.75, AUROC of 0.8910 and AUPRC of 0.9077, respectively (Suppplementary Table S15, see Supplementary Data available online at http://bib.oxfordjournals.org/).

### Performance comparison with different ensemble methods

In this section, we further investigated strategies for integrating the whole sequence model and N-terminal sequence model based on different machine learning techniques, such as mean, logistic regression (LR), k-nearest neighbours (KNN) and SVM. The mean strategy takes the average of the predicted probability of each sub-model as the final result. The LR, KNN and SVM were trained based on the outputs of the sub-models in the training dataset and used as the second level model to generate the final prediction result. The performance results of these different ensemble strategies on 5- and 10-fold cross-validation tests are shown in Figure 4B. We can see that the mean and LR strategies achieved similar AUROC and AUPRC values and outperformed the KNN and SVM strategies. There are several aspects that we need to consider to select the optimal strategy: first, the MCC and F1-score of the mean strategy were much better than that of the LR strategy. On the other hand, the mean strategy had a much lower computational cost than the LR strategy. Second, the LR strategy requires the training process based on the outputs of the sub-models at the second level, which might lead to potential overfitting. In contrast, the mean strategy does not require such a training process. Accordingly, we finally selected the mean strategy to integrate the models based on the above considerations.

**Figure 5:**
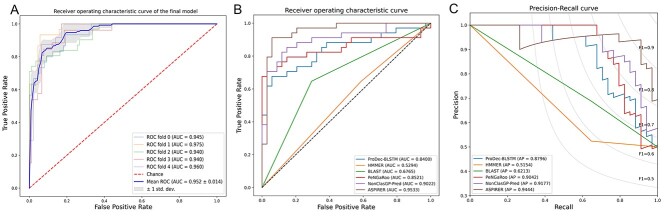
(A) ROC curves of the final model on 5-fold cross-validation; (B) ROC curves and (C) precision-recall curves of ASPIRER and state-of-the-art methods on the independent test.

To assess the performance of the final hybrid model, we performed the 5-fold cross-validation and plotted the receiver-operating characteristic curves in Figure 5A. In addition, we compared the performance of the final model with that of the whole sequence-based XGBoost model and N-terminal-based CNN model in Table 2. As can be seen, the final hybrid model outperformed the two sub-models in terms of multiple performance metrics, with the only exception of Recall. The final model had a slightly lower Recall than the whole sequence-based XGBoost model, presumably because different input features and sampling methods were used by the XGBoost and CNN models. The final model was developed based on the integration of two sub-models, and as such, the Recall of the final model might therefore be affected by the N-terminal sequence-based model. Herein, we are more interested in the AUROC and AUPRC values as these two metrics are reasonable measures of the overall model performance and can reflect the comprehensive performance of different models at varying cutoff thresholds. The results indicate that the combination of the sub-models via the mean ensemble strategy indeed helped improve the performance. In addition, the results also suggest that integrating informative features from the whole amino acid sequence with those extracted from the N-terminal sequence is crucial for identifying NCSPs.

**Table 2:** Performance of the final model and two sub-models on 5-fold cross-validation

Model	Recall	Specificity	Precision	Accuracy	MCC	F1-score	AUROC	AUPRC
XGB	**0.723 ± (0.067)**	0.935 ± (0.015)	0.780 ± (0.038)	0.884 ± (0.016)	0.676 ± (0.050)	0.748 ± (0.042)	0.934 ± (0.013)	0.630 ± (0.048)
CNN	0.646 ± (0.066)	0.928 ± (0.014)	0.737 ± (0.040)	0.860 ± (0.026)	0.601 ± (0.062)	0.688 ± (0.050)	0.909 ± (0.020)	0.784 ± (0.036)
Final model	0.710 ± (0.114)	**0.955 ± (0.025)**	**0.846 ± (0.085)**	**0.896 ± (0.019)**	**0.708 ± (0.055)**	**0.761 ± (0.062)**	**0.952 ± (0.014)**	**0.877 ± (0.039)**

^*^The performance is expressed as mean ± standard deviation and the bold values indicate the best performance.

### Performance comparison with state-of-the-art approaches on the independent test

We compared the performance of ASPIRER with five state-of-the-art approaches by performing the independent test. The compared methods included two machine learning-based methods, PeNGaRoo and NonClasGP-Pred, two popular sequence alignment-based approaches (e.g. PSI-BLAST [53] and HMMER [54]), as well as one remote-homology detection tool (e.g. ProDec-BLSTM [55]). As a result, HMMER was only able to match and identify 23 out of 68 proteins in the independent test dataset. The parameters of the PSI-BLAST and HMMER were set as the default. Table 3 provides the performance results of APSIPER and the five different methods. We can see that ASPIRER achieved the best AUROC and AUPRC; NonClasGP-Pred achieved the best Accuracy, MCC and F1-score, while ProDec-BLSTM achieved the best Precision and Specificity.

**Table 3:** Performance comparison of ASPIRER, baseline models and other existing methods on the independent test

Method	Recall	Specificity	Precision	Accuracy	MCC	F1-score	AUC	AUPRC
BLAST	0.6471	0.7059	0.6875	0.6765	0.3536	0.6667	0.6765	0.6213
HMMER	0.6471	0.4118	0.5238	0.5294	0.0605	0.5789	0.5294	0.5154
ProDec-BLSTM	0.2941	**1.0000**	**1.0000**	0.6471	0.4152	0.4545	0.8400	0.8796
PeNGaRoo	0.8235	0.7353	0.7568	0.7794	0.5610	0.7887	0.8521	0.9042
NonClasGP-Pred	**0.8676**	0.8529	0.8571	**0.8676**	**0.7356**	**0.8696**	0.9019	0.9177
ASPIRER	0.6471	0.9701	0.9565	0.8088	0.6528	0.7719	**0.9533**	**0.9444**

^*^The bold values indicate the best performance.

In this study, we used AUROC and AUPRC as the primary performance metrics to evaluate the model performance. As shown in Table 3, ASPIRER achieved an AUROC of 0.9533 and AUPRC of 0.9444, respectively, clearly outperforming the other methods on the independent test. In particular, the AUROC and AUPRC of ASPIRER were 6 and 3% higher than that of NonClasGP-Pred, respectively, and 12 and 4% higher than those of PeNGaRoo. ProDec-BLSTM achieved the best Specificity and Precision, and the false positive was zero, which indicates those all the predicted positive samples are true positives. The reason is that ProDec-BLSTM can accurately identify homologous NCSPs. However, its other performance metrics were much lower than those of ASPIRER, especially AUROC, AUPRC and Recall. Although HMMER achieved higher accuracy for the matched sequences (i.e. identified homologous sequences in the training dataset), a large portion of the sequences did not have the matched ones, leading to worse performance of HMMER. A possible reason is that HMMER could not efficiently learn from the limited dataset, thereby having a limited predictive capability in identifying such unmatched sequences.

Next, we compared the performance of ASPIRER with NonClasGP-Pred and PeNGaRoo based on the fixed Specificity or Recall values and provided the comparison results in Supplementary Tables S18–S21 (see Supplementary Data available online at http://bib.oxfordjournals.org/). For NonClasGP-Pred, we fixed the same Specificity and similar Recall values (as we could not find the exact same values) as NonClasGP-Pred’s to make the performance comparison, while for PeNGaRoo, we used the same Specificity and Recall values as PeNGaRoo’s. The results show that ASPIRER performed better than NonClasGP-Pred and PeNGaRoo in terms of all performance metrics.

The ROC curves of the state-of-the-art methods, sequence alignment approaches and the remote-homology detection tool are illustrated in Figure 5B. The precision-recall curves of all compared methods and the average precision are shown in Figure 5C. We can see that ASPIRER achieved the overall best performance compared with other methods. In addition, to meet the different requirements, we provide multiple flexible thresholds in the local stand-alone tool of ASPIRER, by which users can adjust the threshold to make the prediction at the preferred precision or recall.

## Conclusions

In this study, we have developed a novel hybrid deep learning-based NCSP predictor, termed ASPIRER, which is based on the integration of a whole sequence-based XGBoost model and an N-terminal sequence-based CNN-based model. More specifically, the two sub-models, respectively, take the whole amino acid sequence and 60 N-terminal residues of NCSPs as the input. Benchmarking experiments on 5-fold cross-validation and independent tests demonstrated that ASPIRER performed better than the existing state-of-the-art approaches and other popular machine learning models. In addition, the results indicate that the N-terminal sequence can provide more informative features than the C-terminal residues for NCSP prediction. It might be that the N-terminal region contains some signals or determinants informative for the secretion of NCSPs. Two critical factors can be attributed to the performance of ASPIRER: (i) it considers both properties from the whole sequence and features from the N-terminal sequences to improve the prediction of NCSPs. (ii) It is developed based on effective integration of two different sub-models. We anticipate that the developed ASPIRER approach can be explored as a valuable tool by the broader research community to accelerate the data-driven discovery of novel putative NCSPs in the future.

**Key Points**

- We propose a hybrid deep learning-based approach, termed ASPIRER, to enable improved prediction of non-classical secreted proteins.
- ASPIRER is developed by integrating an XGBoost model trained with the whole sequence and a CNN model trained with the N-terminal sequence.
- ASPIRER achieves a better performance compared with five state-of-the-art approaches and other popular machine learning algorithms.
- The source code of ASPIRER and the curated datasets are publicly available at https://github.com/yanwu20/ASPIRER/.

## Supplementary Material

Supplementary_material_new_bbac031

## Data Availability

The code and datasets are publicly available at https://github.com/yanwu20/ASPIRER/.

## Notes

**Xiaoyu Wang** received her master’s degree in information technology from The University of Melbourne in 2020. She is currently a research assistant in the Biomedicine Discovery Institute, Monash University and a member of the Bioinformatics and Computational Biomedicine Lab. Her research interests include bioinformatics, computational biomedicine and machine learning.

**Fuyi Li** is currently a Bioinformatics Research Officer in the Department of Microbiology and Immunology, The Peter Doherty Institute for Infection and Immunity, The University of Melbourne, Australia. His research interests are bioinformatics, computational biology, machine learning and data mining.

**Jing Xu** received her BSc and MSc degrees from Nankai University, China. She is currently a PhD student in the Biomedicine Discovery Institute, Monash University and a member of the Bioinformatics and Computational Biomedicine Lab. Her research interests are bioinformatics, computational oncology, machine learning and pattern recognition.

**Jia Rong** is currently a lecturer in the Department of Data Science & AI, Faculty of IT, Monash University. Her research interests are data mining, deep learning, bioinformatics and digital health.

**Geoffrey I. Webb** is a professor in the Faculty of Information Technology and a research director of the Monash Data Futures Institute at Monash University. His research interests include machine learning, data mining, computational biology and user modelling.

**Zongyuan Ge** is an Associate Professor at the Monash e-Research Centre, Monash University. His research interests are AI, computer vision, medical image, robotics and deep learning.

**Jian Li** is a professor and a group leader in the Monash Biomedicine Discovery Institute and Department of Microbiology, Monash University, Australia. He is currently an NHMRC Principal Research Fellow. His research interests include the pharmacology of polymyxins and the discovery of novel, safer polymyxins.

**Jiangning Song** is an Associate Professor and a group leader in the Monash Biomedicine Discovery Institute, Monash University. He is also affiliated with the Monash Data Futures Institute, Monash University. His research interests include bioinformatics, computational biomedicine, machine learning, data mining and pattern recognition.
