# Supplementary material for: ASPIRER: a new computational approach for identifying non-classical secreted proteins based on deep learning
Source: Brief Bioinform. 2022 Feb 17;23(2):bbac031. doi: 10.1093/bib/bbac031 (PMC8921646; doi:10.1093/bib/bbac031)
Supplement: Supplementary_material_new_bbac031 [file supplementary_material_new_bbac031.docx]

**Supplementary Materials**

**ASPIRER: a new computational approach for identifying non-classical secreted proteins based on deep learning**

Xiaoyu Wang^1^, Fuyi Li^2,*^, Jing Xu^1^, Jia Rong^3^, Geoffrey I. Webb^3^, Zongyuan Ge^4,*^, Jian Li^5^, Jiangning Song^1,3,*^

^1^Monash Biomedicine Discovery Institute and Department of Biochemistry and Molecular Biology, Monash University, Melbourne, VIC 3800, Australia;

^2^Department of Microbiology and Immunology, The Peter Doherty Institute for Infection and Immunity, The University of Melbourne, Melbourne, Victoria, Australia;

^3^Department of Data Science and AI, Faculty of Information Technology, Monash University, Melbourne, VIC 3800, Australia;

^4^Monash e-Research Centre and Faculty of Engineering, Monash University, Melbourne, VIC 3800, Australia;

^5^Biomedicine Discovery Institute and Department of Microbiology, Monash University, Melbourne, VIC 3800, Australia.

**Contents**

[Supplemental Methods 4](#_Toc92798592)

[Feature engineering 4](#_Toc92798593)

[Performance assessment 8](#_Toc92798594)

[Analysis of the N-terminal and C-terminal sequence compositions 9](#_Toc92798595)

[Figure S1. T-SNE plots the distributions of the orignial features, feature representation after feature selection and feature representation after applying the SMOTE methd for whole sequence-based XGBoost model. 11](#_Toc92798596)

[Figure S2. Sequence logo representations of the composition difference between the positive and negative samples in the N-and C-terminal sequences. 12](#_Toc92798597)

[Figure S3. Performance comparision of N-terminal sequence-based models in terms of AUROC with different sequence windows on 5-fold cross-validation. 13](#_Toc92798598)

[Table S1. The criterion of amino acid classes for the Composition/Transition/Distribution (CTD) feature descriptors. 14](#_Toc92798599)

[Table S2. Performance comparison of single descriptor models and combined model. 15](#_Toc92798600)

[Table S3. Performance evaluation of different feature selection methods on 5-fold cross-validation. 16](#_Toc92798601)

[Table S4. Performance evaluation of different feature selection methods on the independent test. 17](#_Toc92798602)

[Table S5. Performance comparison of the whole sequence models without FS, with FS, and with FS + SMOTE on 5-fold cross-validation. 18](#_Toc92798603)

[Table S6. Performance evaluation of whole sequence-based models with different processing methods on the independent test. 19](#_Toc92798604)

[Table S7. Performance comparison of the final hybrid model based on WSM without FS, WSM + FS, and WSM + FS + SMOTE on 5-fold cross-validation. 20](#_Toc92798605)

[Table S8. Performance evaluation of different whole sequence models on 5-fold cross-validation. 21](#_Toc92798606)

[Table S9. Performance evaluation of different whole sequence models on the independent test. 22](#_Toc92798607)

[Table S10. Performance evaluation of the final hybrid model with different whole sequence models on 5-fold cross-validation. 23](#_Toc92798608)

[Table S11. Performance evaluation of the N-terminal sequence-based models with different sequence windows on 5-fold cross-validation. 24](#_Toc92798609)

[Table S12. Performance comparison of N-terminal sequence-based models with and without oversampling method on 5-fold cross-validation 25](#_Toc92798610)

[Table S13. Performance comparison of the final hybrid models based on NSM with and without the oversampling method on 5-fold cross-validation. 26](#_Toc92798611)

[Table S14. Performance evaluation of different N-terminal sequence-based models on 5-fold cross-validation. 27](#_Toc92798612)

[Table S15. Performance evaluation of different N-terminal sequence-based models on the independent test. 28](#_Toc92798613)

[Table S16. Performance evaluation of different intergrated models on 5-fold cross-validation. 29](#_Toc92798614)

[Table S17. Performance evaluation of different intergrated models on 10-fold cross-validation. 30](#_Toc92798615)

[Table S18. Performance comparison of ASPIRER and PeNGaRoo with the same Recall value. 31](#_Toc92798616)

[Table S19. Performance comparison of ASPIRER and NonClasGP-Pred with the similar Recall value. 32](#_Toc92798617)

[Table S20. Performance comparison of ASPIRER and PeNGaRoo with the same Specificity value. 33](#_Toc92798618)

[Table S21. Performance comparison of ASPIRER and NonClasGP-Pred with the same Specificity value. 34](#_Toc92798619)

[References: 35](#_Toc92798620)

# Supplemental Methods

# Feature engineering

*Amino Acid Composition (AAC)*

The AAC encoding calculates the frequency of 20 amino acids in the peptide sequence [1]. It can show the basic composition of the amino acid and has been widely used in the bioinformatics field [2-4]. AAC is defined as follow:

$$f\left( x \right)=\frac{N\left( x \right)}{N}, x\in\{A, C, D,\ldots,Y\}$$

where $N\left( x \right)$ is the number of amino acid residues type *x* and *N* denotes the length of the amino acid sequence.

*Composition of k-spaced amino acid pairs (CKSAAP)*

CKSAAP encoding scheme is proposed by Chen. in 2007 [5], and it has been widely used in many bioinformatics prediction tasks, such as functional protein identification [6, 7] and protein functional site prediction [8, 9]. It calculates the frequency of the amino acid pair that spaced by K (K=0, 1, 2, 3 in this study) residues [1, 10] and the output of this descriptor is a 1600-dimensional vector. For example, when *k* = 3, the descriptor is calculated as below:

$${(\frac{N_{AXXXA}}{N_{total}}, \frac{N_{AXXXC}}{N_{total}}, \frac{N_{AXXXD}}{N_{total}},\ldots, \frac{N_{YXXXY}}{N_{total}})}_{400}, X\in\{A, C, D,\ldots,Y\}$$

The $N_{total}$ is the total number of all amino acid pairs spaced by three residues, and the $N_{AXXXA}$ means the number of AA pairs separated by three residues, and X can be any kind of residue.

*Di-Peptide Composition (DPC)*

Dipeptide composition calculates the frequency of dipeptides in protein sequence and generates a 400-dimensional vector. It can be defined as follows:

$$f\left( x,y \right)=\frac{N_{xy}}{N-1}, x,y\in\{A, C, D,\ldots,Y\}$$

where *N* is the number of the total length of the protein or peptide, while the $N_{xy}$ is the number of the *xy* dipeptides.

*Tri-Peptide composition (TPC)*

The TPC encoding is previously used in several studies [11], which generates an 8000-dimensional fixed feature vector. TPC is defined as follows:

$$f\left( x,y,z \right)=\frac{N_{xyz}}{N-2}, x,y,z\in\left\{ A, C, D,\ldots,Y \right\}$$

where the *x*, *y*, and *z* are the amino acid types, and $N_{xyz}$ is the number of the tripeptide *xyz*.

*Dipeptide Deviation from Expected Mean (DDE)*

The Dipeptide Deviation from Expected Mean (DDE) was originally proposed by Saravanan et al. [12], which computed by three parameters: dipeptide composition ($D_{c}$), theoretical mean ($T_{m}$), and theoretical variance ($T_{v}$) [1]. This descriptor The DDE feature descriptor can be calculated as follows:

$$f\left( r,s \right)=\frac{D_{c}\left( r,s \right)-T_{m}(r,s)}{\sqrt{T_{v}(r,s)}}, r,s\in\left\{ A, C, D,\ldots,Y \right\}$$

$D_{c}\left( r,s \right)$ is the dipeptide composition for the $\left( r,s \right)$ dipeptide, and is defined as follows:

$$D_{c}\left( r,s \right)=\frac{N_{rs}}{N-1}, x,y\in\{A, C, D,\ldots,Y\}$$

where *N* is the length of the sequence and $N_{rs}$ is the number of the dipeptide *rs*. *r* and *s* denote the amino acid types. $T_{m}(r,s)$ is given as:

$$T_{m}\left( r,s \right)=\frac{C_{r}}{C_{N}} \times\frac{C_{s}}{C_{N}}$$

where the $C_{r}$ and $C_{s}$ are the numbers of the codons corresponding to the amino acid *r* and amino acid *s*. For example, the methionine (M) only has one codon, so the $C_{M}$ is 1. $C_{N}$ is the total number of codons for all the 20 amino acid types (i.e., 61). Moreover, $T_{v}$ is the theoretical mean, which is defined as:

$$T_{v}\left( r,s \right)=\frac{T_{m}(r,s)(1-T_{m}\left( r,s \right))}{N-1}$$

*Composition/Transition/Distribution (CTD)*

The CTD feature descriptors [13] describe the physicochemical properties and structural properties of the amino acid sequence. 13 types of physiochemical properties, including hydrophobicity, Normalized van der waals volume, polarity, charge, secondary structure, solvent accessibility and so forth, were used as the criterion of the amino acid group (**Table S1**) [14].

*Composition (CTDC)*

The CTDC descriptor characterizes the composition of the amino acid sequence from three physicochemical groups (**Table S1**), and is defined as follows:

$$f\left( r \right)=\frac{N(r)}{N}, r\in\left\{ polar,neutral,hydrophobic \right\}$$

The $N\left( r \right)$ is the number of particular amino acid residue type *r* and *r* belongs to one physicochemical group. *N* is the number of the amino acids (i.e., the length of the sequence).

*Transition (CTDT)*

The CTDT descriptor measures the percentage frequency of the particular amino acids which followed by amino acid of another physicochemical group. The CTDT descriptor is calculated as:

$$f\left( r,s \right)=\frac{N\left( r,s \right)+N\left( s,r \right)}{N-1},$$

$$r,s\in\left\{ \left( polar,neutral \right),\left( neutral,hydrophobic \right),(hydrophobic,polar) \right\}$$

where $N\left( r,s \right)$ denotes the number of the dipeptide that contains two amino acids *r*,*s*, and the *r* and *s* are in different groups.

*Distribution (CTDD)*

The CTDD descriptor describes the distribution of each physicochemical properties in the sequence. It contains five values for each physicochemical group corresponding to the position fractions of the whole sequence where the first, 25%, 50%, 75% and 100% residues of a certain group are located, respectively.

*Conjoint Triad (CTriad)*

The CTriad descriptor represents the properties of amino acids and their vicinal amino acids, and three continuous amino acids can be recognized as a unit. The amino acids are classified into seven classes (g1: AGV, g2: ILFP, g3: YMTS, g4: HNQW, g5: RK, g6: DE, g7: C). It is a 343-dimensional vector, and each vector is the frequency of the conjoint triad appearing in the peptides sequence.

*Pseudo Position-Specific Score Matrix (Pse-PSSM)*

Pse-PSSM is an evolution-based feature calculated from the PSSM, which is generated by PSI-BLAST search against the Uniref50 database. Compared with PSSM, Pse-PSSM retains the sequence-order information. The Pse-PSSM encoding method was proposed by Chou *et al*. [15], and it returns a 40-dimensional vector, which is represented by [16]:

$$P_{Pse-PSSM}^{\xi}=\left[ \bar{\mathbb{E}_{1}} \bar{\mathbb{E}_{2}} \ldots\bar{\mathbb{E}_{j}} G_{1}^{\xi} G_{2}^{\xi}\ldots G_{20}^{\xi} \right]^{T}$$

$\bar{\mathbb{E}_{j}}$ represent the average score of the amino acid residues that mutated to amino acid residues *j*, and $G_{j}^{\xi}$means the correlation factor by coupling the $\xi$-th ($\xi$ =1) most contiguous PSSM score for the amino acid residue *j*. The formulas of $\bar{\mathbb{E}_{j}}$and $G_{j}^{\xi}$ are given below:

$$\mathbb{E}_{i\to j}= \frac{\mathbb{E}_{i\to j}^{0}-\frac{1}{20}\sum_{k=1}^{20} \mathbb{E}_{i\to k}^{0}}{\sqrt{\frac{1}{20}\sum_{m=1}^{20} {(\mathbb{E}_{i\to m}^{0}-\frac{1}{20}\sum_{k=1}^{20} \mathbb{E}_{i\to k}^{0})}^{2}}}, (i=1, 2,\ldots, L;j=1, 2, \ldots, 20)$$

$$\bar{\mathbb{E}_{j}}=\frac{1}{L}\sum_{i=1}^{L} \mathbb{E}_{i\to j}, (j=1, 2, \ldots, 20)$$

$$G_{j}^{\xi}=\frac{1}{L-\xi}\sum_{i=1}^{L-\xi} {(\mathbb{E}_{i\to j}-\mathbb{E}_{(i+\xi)\to j})}^{2}, (j=1, 2, \ldots, 20; \xi<L)$$

where $\mathbb{E}_{i\to j}$ represent the average score of the residue in the *i-*th position mutated to the amino acid residue *j*, while $\mathbb{E}_{i\to m}^{0}$ means the original score generated by PSI-BLAST.

*Grouped amino acid composition (GAAC)*

GAAC encoding scheme calculates the frequency of the amino acid types by their physicochemical properties, and it is the variation of AAC descriptor. The amino acids are classified into five classes: the aliphatic (group 1: GAVLMI), aromatic (group 2: FYW), positive (group 3: KRH), and negative charged groups (group 4: DE) and uncharged group (group 5: STCPNQ).

$$f\left( c \right)=\frac{N\left( c \right)}{N}, c\in\{c1, c2, c3, c4,c5\}$$

$$N\left( C \right)= \sum N(t), t\in c$$

where $N\left( c \right)$ is the number of amino acids in class $c$, and $t$ is the amino acid type that belongs to class $c$. *N* denotes the length of the protein.

*Grouped Di-Peptide Composition (GDPC)*

The GDPC encoding is similar to DPC in that they both calculate the frequency of amino acid pairs, while the amino acids are categorized into five classes. GDPC returns a 25-dimensional vector. The criterion of amino acid classes is the same as GAAC. It can be defined as:

$$f\left( r,s \right)=\frac{N\left( r,s \right)}{N}, r,s\in\{c1, c2, c3, c4,c5\}$$

$$N\left( r,s \right)= \sum N(t), t \in r,s$$

where the $N\left( r,s \right)$ is the number of amino acid pair $\left( r,s \right)$ while $t$ is the type of amino acid pairs.

*Moran correlation*

The Moran correlation feature descriptor describes the distribution of physicochemical properties of amino acids. Amino acid indices represent the physicochemical properties of amino acids and can be extracted from the AAindex Database [17]. There are a total of 531 different amino acid indices included in this descriptor. The calculation of Moran autocorrelation descriptor [1, 18]is defined as follows:

$$f\left( d \right)= \frac{\frac{1}{N-d}\sum_{i=1}^{N-d} \left( P_{i}-\bar{P} \right)\left( P_{i+d}-\bar{P} \right)}{\frac{1}{N}\sum_{i=1}^{N} \left( P_{i}-\bar{P} \right)^{2}}, (d=1, 2, 3\ldots, nlag)$$

where *d* represents the lag of autocorrelation, and *nlag* is the maximum lag (*nlag* = 2) in this study. $P_{i}$ is the properties of the amino acid residues at the position *i* while $P_{i+d}$ means the properties of residues at the position *i+d*. $\bar{P}$is the average values of all considered properties *P* in the whole sequence of the length *N*.

# Performance assessment

Eight performance metrics are commonly used to evaluate the performance of the models, including Precision, Recall, Accuracy, Matthew’s correlation coefficient (MCC), F1-score, Specificity, the area under the receiver operating characteristic (ROC) curve (AUROC) and the area under the precision-recall (PR) curve (AUPRC). These performance metrics can be calculated as follows:

$$Precision=\frac{TP}{TP+FP}$$

$$Recall=\frac{TP}{TP+FN}$$

$$Accuracy=\frac{TP+TN}{TP+TN+FP+FN}$$

$$MCC=\frac{TP*TN-FP*FN}{\sqrt{(TP+FP)(TP+FN)(TN+FP)(TN+FN)}}$$

$$F1-score=2*\frac{(precision*recall)}{precision+recall}$$

$$FPR=\frac{FP}{TN+FP}$$

where *TN*, *TP*, *FN*, and *FP* denote the numbers of true negatives (i.e. correctly predicted non-NCSPs), true positives (i.e. correctly predicted NCSPs), false negatives (i.e. incorrectly predicted non-NCSPs), and false positives (i.e. incorrectly predicted NCSPs), respectively. The ROC curve plots the false positive rate (FPR) as the x-axis and the true positive rate (TPR) as the y-axis. In addition, the Precision-Recall curve uses the precision as the y-axis while the recall as the x-axis. Accordingly, the area under the Precision-Recall curves is also used as the primary metric to evaluate the performance of different models in cases where the dataset is highly imbalanced. Since the dataset is imbalanced, the accuracy cannot correctly evaluate the model’s performance, and the AUROC is used as the primary evaluation matrix.

K-fold cross-validation is one of the most commonly used methods for assessing the performance of machine learning models [19, 20]. In this study, to compare the model’s performance, we performed K-fold (K=5) cross-validation to assess the model performance. In addition, we also conducted the independent test to further compare and evaluate the model performance.

**Supplemental results**

# Analysis of the N-terminal and C-terminal sequence compositions

Previous studies have shown that the deletion of even a single N-terminal residue could result in the inhibition of the secretion of NCSPs, which indicates the N-terminal region is essential for the NCSP secretion. Furthermore, the truncation of five C-terminal residues also led to secretion inhibition [21]. These studies indicate that both the N- and C- terminal residues are critical for the secretion of NCSPs. In addition, the protein length of NCSPs varies from 67 to thousands, and the less relevant area might hide some functional regions. Thus, to help find the more relevant functional regions of non-classical protein, we compared the composition difference between 60 N-terminal and C-terminal residues by generating the sequence logo representations in **Figure S2** using the Two Sample logo software [22]. The statistically significant difference in the amino acid composition of each position is displayed in **Figure S2**. The statistical analysis was carried by t-test with p value < 0.5. From **Figure S2**, we can see that the composition of N-terminal residues between positive samples and negative samples is more significantly different than that of the C-terminal residues. This indicates that the N-terminal residues might be more helpful for the identification of the non-classical secreted protein, which is consistent with previous studies. In addition, another observation is that positive samples are enriched in Alanine (A), Glycine (G), Valine (V), Threonine (T), and less Lysine (K), Leucine (L) compared with negative samples. An interesting observation is that at the position 2 of the N-terminal residues, the composition of the positive samples appeared to be significantly different from that of the negative samples. It might consistent with Zhao *et al*.’s work [21], when two or more N-terminal residues were deleted, the RDPE (D-psicose 3-epimerase from *Ruminococcus sp.* 5_1_39BFAA), a confirmed NCSPs, cannot be detected in extracellular environment. In addition, previous studies have shown that the NCSPs were detected as multimers during the secretion process and the termination area might help maintain the necessary structure of NCSPs for secretion [21, 23].Based on this information and our result, we assume that N-terminal sequences can help to identify some properties of NCSPs.


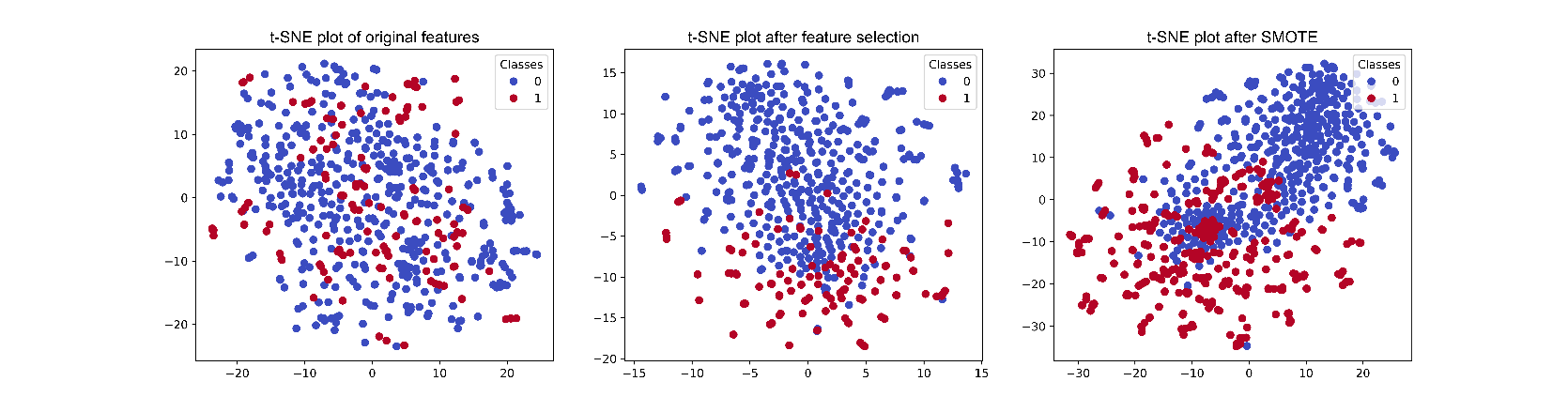


Figure S1. T-SNE plots the distributions of the orignial features, feature representation after feature selection and feature representation after applying the SMOTE methd for whole sequence-based XGBoost model. ‘0’ represents the negative samples while ‘1’ denotes positive samples, i.e. non-classical secreted proteins.

**
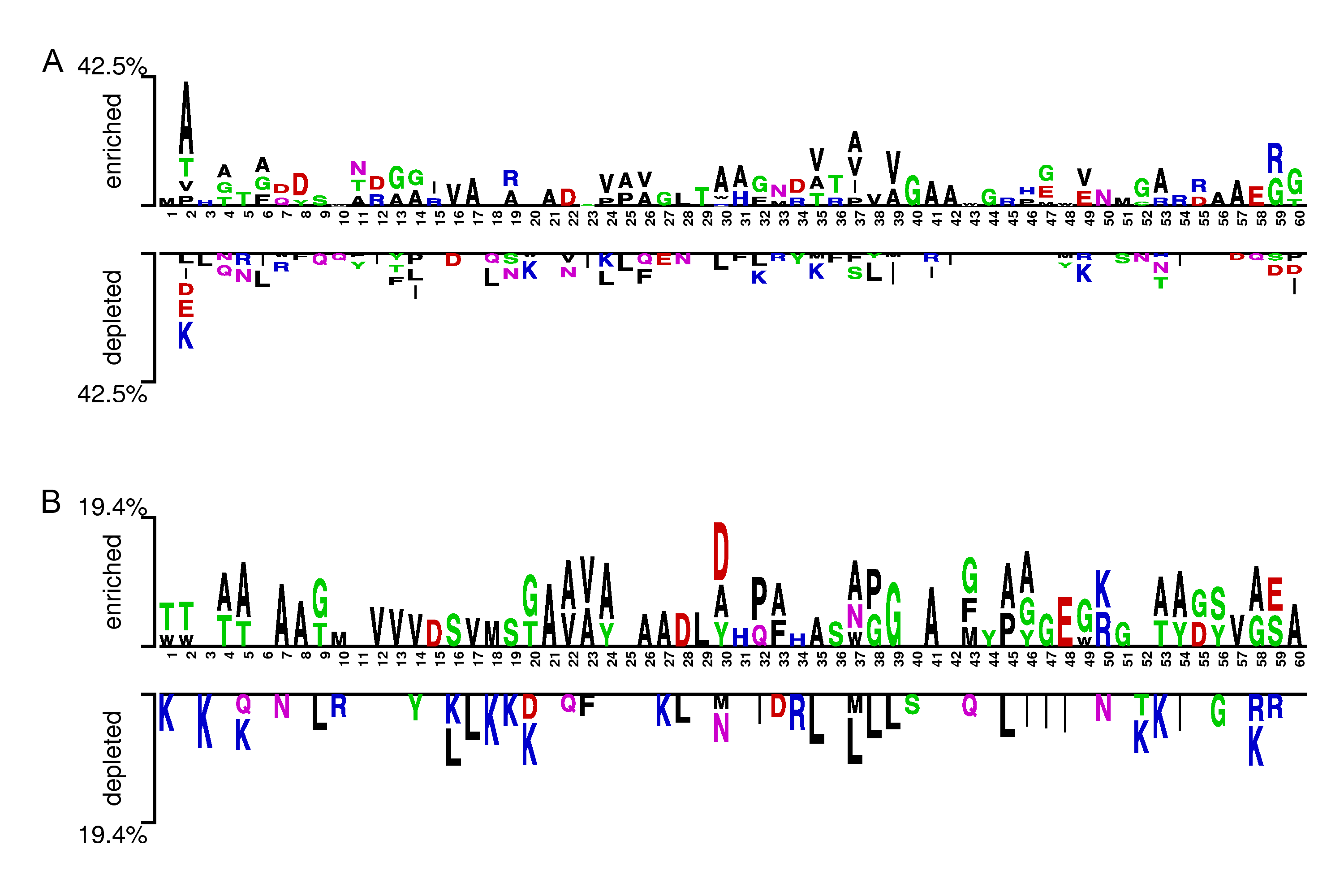
**

Figure S2. Sequence logo representations of the composition difference between the positive and negative samples in the N-and C-terminal sequences. (A) Sequence logo of 60 N-terminal amino acid residues; (B) Sequence logo of 60 C-terminal amino acid sequence residues.


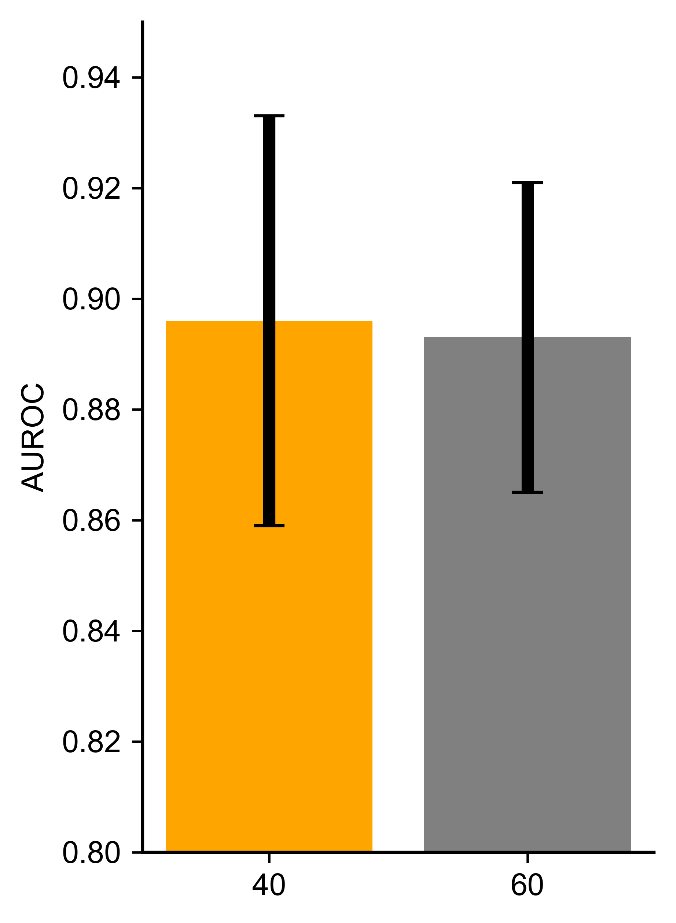


# Figure S3. Performance comparision of N-terminal sequence-based models in terms of AUROC with different sequence windows on 5-fold cross-validation.

# Table S1. The criterion of amino acid classes for the Composition/Transition/Distribution (CTD) feature descriptors.

| **Attribute** | **Division** | | |
| --- | --- | --- | --- |
| Hydrophobicity_PRAM900101 | Polar: RKEDQN | Neutral: GASTPHY | Hydrophobicity: CLVIMFW |
| Hydrophobicity_ARGP820101 | Polar:  QSTNGDE | Neutral: RAHCKMV | Hydrophobicity: LYPFIW |
| Hydrophobicity_ZIMJ680101 | Polar:  QNGSWTDERA | Neutral: HMCKV | Hydrophobicity: LPFYI |
| Hydrophobicity_PONP930101 | Polar:  KPDESNQT | Neutral: GRHA | Hydrophobicity:  YMFWLCVI |
| Hydrophobicity_CASG920101 | Polar:  KDEQPSRNTG | Neutral: AHYMLV | Hydrophobicity: FIWC |
| Hydrophobicity_ENGD860101 | Polar:  RDKENQHYP | Neutral :SGTAW | Hydrophobicity: CVLIMF |
| Hydrophobicity_FASG890101 | Polar: KERSQD | Neutral: NTPG | Hydrophobicity:  AYHWVMFLIC |
| Normalized van der Waals volume | Volume range:  0-2.78  GASTPD | Volume range:  0-2.78  GASTPD | Volume range: 4.03-8.08  MHKFRYW |
| Polarity | Polarity value:  4.9-6.2  LIFWCMVY | Polarity value: 8.0-9.2  PATGS | Polarity value: 10.4-13.0  HQRKNED |
| Polarizability | Polarizability  value: 0-1.08  GASDT | Polarizability value:  0.128-120.186  GPNVEQIL | Polarizability value: 0.219-0.409  KMHFRYW |
| Charge | Positive: KR | Neutral: ANCQGHILMFPSTWYV | Negative: DE |
| Secondary structure | Helix: EALMQKRH | Strand: VIYCWFT | Coil: GNPSD |
| Solvent accessibility | Buried: ALFCGIVW | Exposed: PKQEND | Intermediate: MPSTHY |

# Table S2. Performance comparison of single descriptor models and combined model.

| Feature | Recall | Specificity | Precision | Accuracy | MCC | F1-score | AUROC | AUPRC |
| --- | --- | --- | --- | --- | --- | --- | --- | --- |
| AAC | **0.688±(0.032)** | 0.946±(0.024) | 0.808±(0.070) | 0.884±(0.018) | 0.672±(0.048) | **0.741±(0.032)** | 0.924±(0.009) | 0.631±(0.048) |
| CKSAAP | 0.574±(0.023) | 0.957±(0.015) | 0.814±(0.051) | 0.865±(0.009) | 0.606±(0.028) | 0.672±(0.018) | 0.934±(0.007) | 0.569±(0.026) |
| Pse-PSSM | 0.681±(0.039) | 0.933±(0.033) | 0.773±(0.085) | 0.872±(0.025) | 0.643±(0.060) | 0.720±(0.041) | 0.934±(0.018) | 0.602±(0.058) |
| EEDP | 0.617±(0.089) | 0.948±(0.018) | 0.791±(0.073) | 0.869±(0.031) | 0.619±(0.094) | 0.691±(0.078) | 0.922±(0.018) | 0.584±(0.085) |
| CTDC | 0.667±(0.032) | 0.933±(0.016) | 0.761±(0.041) | 0.869±(0.012) | 0.629±(0.031) | 0.710±(0.023) | 0.934±(0.012) | 0.587±(0.029) |
| CTDT | 0.639±(0.066) | 0.919±(0.023) | 0.717±(0.063) | 0.852±(0.025) | 0.582±(0.070) | 0.674±(0.056) | 0.918±(0.009) | 0.547±(0.063) |
| CTDD | 0.368±(0.103) | 0.966±(0.017) | 0.785±(0.130) | 0.823±(0.023) | 0.449±(0.100) | 0.492±(0.104) | 0.860±(0.050) | 0.443±(0.069) |
| CTriad | 0.539±(0.033) | 0.946±(0.021) | 0.768±(0.069) | 0.848±(0.014) | 0.554±(0.040) | 0.631±(0.026) | 0.912±(0.012) | 0.524±(0.035) |
| DDE | 0.623±(0.069) | 0.944±(0.029) | 0.789±(0.088) | 0.867±(0.025) | 0.619±(0.071) | 0.692±(0.055) | 0.923±(0.016) | 0.582±(0.064) |
| GAAC | 0.482±(0.081) | 0.904±(0.031) | 0.615±(0.106) | 0.802±(0.038) | 0.422±(0.115) | 0.539±(0.090) | 0.827±(0.046) | 0.428±(0.083) |
| GDPC | 0.510±(0.055) | 0.933±(0.027) | 0.712±(0.098) | 0.831±(0.029) | 0.502±(0.086) | 0.593±(0.063) | 0.858±(0.026) | 0.484±(0.065) |
| Moran | 0.234±(0.050) | **0.984±(0.015)** | **0.852±(0.129)** | 0.804±(0.014) | 0.375±(0.059) | 0.362±(0.061) | 0.800±(0.042) | 0.382±(0.039) |
| DPC | 0.581±(0.068) | 0.944±(0.020) | 0.769±(0.071) | 0.857±(0.023) | 0.582±(0.072) | 0.660±(0.060) | 0.923±(0.018) | 0.549±(0.065) |
| TPC | 0.432±(0.054) | 0.964±(0.021) | 0.799±(0.098) | 0.836±(0.023) | 0.504±(0.080) | 0.559±(0.063) | 0.892±(0.053) | 0.484±(0.064) |
| All feature | 0.674±(0.024) | 0.953±(0.016) | 0.822±(0.053) | **0.886±(0.010)** | **0.674±(0.031)** | 0.739±(0.020) | **0.937±(0.0014)** | **0.632±(0.033)** |

*Performance is expressed as mean ± standard deviation. “All feature” refers to the model that combines all features without feature selection.

# Table S3. Performance evaluation of different feature selection methods on 5-fold cross-validation.

| Method | Recall | Specificity | Precision | Accuracy | MCC | F1-score | AUROC | AUPRC |
| --- | --- | --- | --- | --- | --- | --- | --- | --- |
| Chi2 | 0.666±(0.055) | **0.951±(0.029)** | **0.821±(0.088)** | **0.882±(0.022)** | **0.666±(0.064)** | 0.732±(0.047) | **0.939±(0.009)** | **0.626±(0.064)** |
| L1-based | **0.695±(0.038)** | 0.937±(0.029) | 0.786±(0.076) | 0.879±(0.020) | 0.661±(0.051) | **0.735±(0.035)** | 0.936±(0.013) | 0.619±(0.054) |
| Tree-based | 0.653±(0.039) | 0.949±(0.029) | 0.812±(0.086) | 0.877±(0.017) | 0.652±(0.047) | 0.720±(0.029) | 0.934±(0.010) | 0.611±(0.049) |

*Performance is expressed as mean ± standard deviation while the bold values indicate the best performance.

# Table S4. Performance evaluation of different feature selection methods on the independent test.

| Method | Recall | Specificity | Precision | Accuracy | MCC | F1-score | AUROC | AUPRC |
| --- | --- | --- | --- | --- | --- | --- | --- | --- |
| Chi2 | 0.6176 | 0.9118 | 0.8750 | 0.7647 | 0.5539 | 0.7241 | **0.8772** | 0.8989 |
| L1-based | 0.6176 | 0.9412 | 0.9130 | 0.7794 | 0.5906 | 0.7368 | 0.8469 | **0.8911** |
| Tree-based | **0.6765** | **0.9706** | **0.9583** | **0.8235** | **0.6770** | **0.7931** | 0.8599 | 0.8885 |

* The bold values indicate the best performance.

# Table S5. Performance comparison of the whole sequence models without FS, with FS, and with FS + SMOTE on 5-fold cross-validation.

| Method | Recall | Specificity | Precision | Accuracy | MCC | F1-score | AUROC | AUPRC |
| --- | --- | --- | --- | --- | --- | --- | --- | --- |
| WSM (  w/o FS) | 0.674±(0.024) | **0.953±(0.016)** | **0.822±(0.053)** | 0.886±(0.010) | 0.674±(0.031) | 0.739±(0.020) | 0.937±(0.0014**)** | **0.632±(0.033)** |
| WSM (  With FS) | 0.666±(0.055) | 0.951±(0.029) | 0.821±(0.088) | **0.882±(0.022)** | 0.666±(0.064) | 0.732±(0.047) | **0.939±(0.009)** | 0.626±(0.064) |
| WSM (  FS+SMOTE) | **0.723±(0.067)** | 0.935±(0.015) | 0.780±(0.038) | 0.884±(0.016) | **0.676±(0.050)** | **0.748±(0.042)** | 0.934±(0.013) | 0.630±(0.048) |

*The bold values indicate the best performance. “WSM” the means whole sequence model “FS” means feature selection. The performance was evaluated based on the XGBoost model with all features.

# Table S6. Performance evaluation of whole sequence-based models with different processing methods on the independent test.

| Method | Recall | Specificity | Precision | Accuracy | MCC | F1-score | AUROC | AUPRC |
| --- | --- | --- | --- | --- | --- | --- | --- | --- |
| WSM (  w/o FS) | **0.7058** | **0.9412** | **0.9231** | **0.8235** | **0.6658** | **0.8000** | 0.8901 | 0.9036 |
| WSM (  With FS) | 0.5882 | **0.9412** | 0.9090 | 0.7647 | 0.5658 | 0.7143 | 0.8868 | 0.8837 |
| WSM (  FS+SMOTE) | 0.6471 | **0.9412** | 0.9167 | 0.7941 | 0.6155 | 0.7586 | **0.9066** | **0.9157** |

*The bold values indicate the best performance. “WSM” means whole sequence model. “FS” means feature selection. The performance was evaluated based on the XGBoost model with all features.

# Table S7. Performance comparison of the final hybrid model based on WSM without FS, WSM + FS, and WSM + FS + SMOTE on 5-fold cross-validation.

| Methods | Recall | Specificity | Precision | Accuracy | MCC | F1-score | AUROC | AUPRC |
| --- | --- | --- | --- | --- | --- | --- | --- | --- |
| Final model  (WSM w/o FS) | 0.679±(0.114) | **0.960±(0.021)** | **0.847±(0.076)** | 0.893±(0.020) | 0.691±(0.070) | 0.745±(0.072) | 0.951±(0.010) | 0.868±(0.044) |
| Final model (WSM+FS) | 0.688±(0.082) | 0.951±(0.021) | 0.822±(0.074) | 0.888±(0.012) | 0.680±(0.042) | 0.742±(0.045) | 0.945±(0.014) | 0.856±(0.054) |
| Final model (WSM+FS+SMOTE) | **0.718±(0.118)** | 0.953±(0.025) | 0.841±(0.087) | **0.896±(0.020)** | **0.709±(0.056)** | **0.763±(0.062)** | **0.952±(0.014)** | **0.877±(0.039)** |

*The bold values indicate the best performance. “WSM” means whole sequence model. “FS” means feature selection.

# Table S8. Performance evaluation of different whole sequence models on 5-fold cross-validation.

| Method | Recall | Specificity | Precision | Accuracy | MCC | F1-score | AUROC | AUPRC |
| --- | --- | --- | --- | --- | --- | --- | --- | --- |
| XGB | 0.723±(0.067) | 0.935±(0.015) | 0.780±(0.038) | 0.884±(0.016) | 0.676±(0.050) | 0.748±(0.042) | 0.934±(0.013) | 0.630±(0.048) |
| SVM | 0.688±(0.068) | **0.953±(0.026)** | **0.825±(0.092)** | 0.889±(0.032) | 0.685±(0.095) | 0.750±(0.074) | **0.940±(0.013)** | 0.648±(0.097) |
| RF | 0.708±(0.071) | 0.926±(0.021) | 0.753±(0.062) | 0.874±(0.026) | 0.648±(0.074) | 0.729±(0.058) | 0.928±(0.021) | 0.606±(0.071) |
| CNN | **0.780±(0.052)** | 0.935±(0.026) | 0.794±(0.072) | **0.898±(0.030)** | **0.720±(0.079)** | **0.787±(0.058)** | 0.940±(0.033) | **0.843±(0.098)** |

*Performance is expressed as mean ± standard deviation while the bold values indicate the best performance.

# Table S9. Performance evaluation of different whole sequence models on the independent test.

| Method | Recall | Specificity | Precision | Accuracy | MCC | F1-score | AUROC | AUPRC |
| --- | --- | --- | --- | --- | --- | --- | --- | --- |
| XGB | **0.6471** | 0.9412 | 0.9167 | **0.7941** | **0.6155** | **0.7586** | **0.9066** | **0.9157** |
| SVM | 0.5000 | **0.9706** | **0.9444** | 0.7353 | 0.5333 | 0.6538 | 0.8045 | 0.8560 |
| RF | **0.6471** | 0.9118 | 0.8800 | 0.7794 | 0.5795 | 0.7458 | 0.8594 | 0.8909 |
| CNN | 0.6176 | 0.9118 | 0.8750 | 0.7647 | 0.5539 | 0.7241 | 0.8097 | 0.8522 |

* The bold values indicate the best performance.

# Table S10. Performance evaluation of the final hybrid model with different whole sequence models on 5-fold cross-validation.

| Method | Recall | Specificity | Precision | Accuracy | MCC | F1-score | AUROC | AUPRC |
| --- | --- | --- | --- | --- | --- | --- | --- | --- |
| XGB | 0.718±(0.118) | 0.953±(0.025) | **0.841±(0.087)** | 0.896±(0.020) | 0.709±(0.056) | 0.763±(0.062) | **0.952±(0.014)** | **0.877±(0.039)** |
| SVM | 0.665±(0.083) | **0.964±(0.014)** | 0.853±(0.067) | 0.893±(0.017) | 0.688±(0.067) | 0.743±(0.062) | 0.950±(0.011) | 0.862±(0.048) |
| RF | 0.661±(0.080) | 0.958±(0.015) | 0.831±(0.063) | 0.886±(0.016) | 0.671±(0.055) | 0.731±(0.053) | 0.946±(0.008) | 0.864±(0.019) |
| CNN | **0.790±(0.113)** | 0.946±(0.029) | 0.827±(0.088) | **0.908±(0.030)** | **0.747±(0.088)** | **0.800±(0.074)** | 0.930±(0.021) | 0.841±(0.054) |

*Performance is expressed as mean ± standard deviation and the bold values indicate the best performance.

# Table S11. Performance evaluation of the N-terminal sequence-based models with different sequence windows on 5-fold cross-validation.

| Method | Recall | Specificity | Precision | Accuracy | MCC | F1_score | AUROC | AUPRC |
| --- | --- | --- | --- | --- | --- | --- | --- | --- |
| CNN (40) | **0.673±(0.082**) | 0.924±(0.026) | 0.740±(0.063) | 0.864±(0.025) | **0.617±(0.073)** | **0.702±(0.058)** | **0.896±(0.037)** | **0.772±(0.083)** |
| CNN (60) | 0.638±(0.048) | **0.937±(0.020)** | **0.765±(0.063)** | **0.865±(0.024)** | 0.614±(0.067) | 0.695±(0.051) | 0.893±(0.028) | 0.762±(0.063) |

*The performance is shown as mean ± standard deviation and the bold values indicate the best performance.

# Table S12. Performance comparison of N-terminal sequence-based models with and without oversampling method on 5-fold cross-validation

| Method | Recall | Specificity | Precision | Accuracy | MCC | F1_score | AUROC | AUPRC |
| --- | --- | --- | --- | --- | --- | --- | --- | --- |
| NSM (w/o oversampling) | 0.638±(0.048) | **0.937±(0.020)** | **0.765±(0.063)** | **0.865±(0.024)** | **0.614±(0.067)** | **0.695±(0.051)** | 0.893±(0.028) | 0.762±(0.063) |
| NSM (oversampling) | **0.646±(0.066)** | 0.928±(0.014) | 0.737±(0.040) | 0.860±(0.026) | 0.601±(0.062) | 0.688±(0.050) | **0.909±(0.020)** | **0.784±(0.036)** |

*The performance is shown as mean ± standard deviation and the bold values indicate the best performance.

“NSM” means the N-terminal sequence-based models. The performance was evaluated based on the CNN model.

# Table S13. Performance comparison of the final hybrid models based on NSM with and without the oversampling method on 5-fold cross-validation.

| Method | Recall | Specificity | Precision | Accuracy | MCC | F1_score | AUROC | AUPRC |
| --- | --- | --- | --- | --- | --- | --- | --- | --- |
| Final model (NSM w/o oversampling) | 0.605±(0.079) | **0.989±(0.015)** | **0.949±(0.063)** | 0.896±(0.029) | 0.703±(0.072) | 0.736±(0.066) | 0.945±(0.025) | **0.891±(0.041)** |
| Final model (NSM oversampling) | **0.710±(0.114)** | 0.955±(0.025) | 0.846±(0.085) | **0.896±(0.019)** | **0.708±(0.055)** | **0.761±(0.062)** | **0.952±(0.014)** | 0.877±(0.039) |

*The performance is shown as mean ± standard deviation and the bold values indicate the best performance. “NSM” means the N-terminal sequence-based models. The performance was evaluated based on the CNN model.

# Table S14. Performance evaluation of different N-terminal sequence-based models on 5-fold cross-validation.

| Method | Recall | Specificity | Precision | Accuracy | MCC | F1_score | AUROC | AUPRC |
| --- | --- | --- | --- | --- | --- | --- | --- | --- |
| XGB | 0.563±(0.094) | 0.930±(0.027) | 0.730±(0.068) | 0.842±(0.011) | 0.542±(0.025) | 0.625±(0.046) | 0.876±(0.018) | 0.510±(0.026) |
| SVM | 0.291±(0.081) | **0.980±(0.015)** | **0.820±(0.105)** | 0.814±(0.026) | 0.412±(0.099) | 0.425±(0.098) | **0.913±(0.020)** | 0.414±(0.077) |
| RF | 0.451±(0.088) | 0.960±(0.022) | 0.798±(0.104) | 0.837±(0.022) | 0.511±(0.050) | 0.565±(0.059) | 0.896±(0.017) | 0.487±(0.034) |
| CNN | **0.646±(0.066)** | 0.928±(0.014) | 0.737±(0.040) | **0.860±(0.026)** | **0.601±(0.062)** | **0.688±(0.050)** | 0.909±(0.020) | **0.784±(0.036)** |

*The performance is shown as mean ± standard deviation and the bold values indicate the best performance.

# Table S15. Performance evaluation of different N-terminal sequence-based models on the independent test.

| Method | Recall | Specificity | Precision | Accuracy | MCC | F1-score | AUROC | AUPRC |
| --- | --- | --- | --- | --- | --- | --- | --- | --- |
| XGB | 0.4706 | 0.9412 | 0.8889 | 0.7059 | 0.4667 | 0.6154 | 0.7785 | 0.8280 |
| SVM | 0.3235 | **1.0000** | **1.0000** | 0.6618 | 0.4393 | 0.4889 | 0.7638 | 0.8331 |
| RF | 0.3824 | **1.0000** | **1.0000** | 0.6912 | 0.4862 | 0.5532 | 0.8002 | 0.8439 |
| CNN | **0.6176** | 0.9706 | 0.9545 | **0.7941** | **0.6287** | **0.7500** | **0.8910** | **0.9077** |

* The bold values indicate the best performance.

# Table S16. Performance evaluation of different intergrated models on 5-fold cross-validation.

| Method | Recall | Specificity | Precision | Accuracy | MCC | F1-score | AUROC | AUPRC |
| --- | --- | --- | --- | --- | --- | --- | --- | --- |
| Mean | 0.710±(0.114) | 0.955±(0.025) | 0.846±(0.085**)** | 0.896±(0.019) | 0.708±(0.055) | 0.761±(0.062) | **0.952±(0.014)** | 0.877±(0.039) |
| LR | 0.641±(0.148) | **0.964±(0.018)** | **0.863±(0.071)** | 0.886±(0.026) | 0.672±(0.074) | 0.718±(0.099) | 0.952±(0.015) | **0.879±(0.033**) |
| KNN | **0.781±(0.076)** | 0.953±(0.026) | 0.848±(0.085) | **0.911±(0.009)** | **0.756±(0.030)** | **0.806±(0.031)** | 0.868±(0.028) | 0.715±(0.038) |
| SVM | 0.745±(0.087) | 0.955±(0.025) | 0.849±(0.084) | 0.905±(0.017) | 0.733±(0.050) | 0.786±(0.046) | 0.949±(0.023) | 0.879±(0.035) |

*Performance is expressed as mean ± standard deviation while the bold values indicate the best performance.

# Table S17. Performance evaluation of different intergrated models on 10-fold cross-validation.

| Method | Recall | Specificity | Precision | Accuracy | MCC | F1-score | AUROC | AUPRC | |
| --- | --- | --- | --- | --- | --- | --- | --- | --- | --- |
| Mean | 0.695±(0.102) | 0.949±(0.037) | 0.821±(0.119) | 0.887±(0.021) | 0.683±(0.074) | 0.742±(0.067) | **0.948±(0.025)** | 0.864±(0.076) | |
| LR | 0.639±(0.137) | **0.960±(0.029)** | **0.844±(0.107)** | 0.882±(0.025) | 0.661±(0.086) | 0.713±(0.086) | 0.948±(0.026) | **0.865±(0.078)** |  |
| KNN | **0.752±(0.102)** | 0.947±(0.041) | 0.828±(0.138) | 0.899±(0.032) | 0.723±(0.100) | 0.778±(0.080) | 0.851±(0.047) | 0.688±(0.081) |  |
| SVM | 0.746±(0.115) | 0.953±(0.033) | 0.844±(0.109) | **0.903±(0.019)** | **0.730±(0.067)** | **0.780±(0.062)** | 0.944±(0.025) | 0.870±(0.058) |  |

*Performance is shown as mean ± standard deviation while the bold values indicate the best performance.

# Table S18. Performance comparison of ASPIRER and PeNGaRoo with the same Recall value.

| Tool | Recall | Specificity | Precision | Accuracy | MCC | F1-score | AUROC | AUPRC |
| --- | --- | --- | --- | --- | --- | --- | --- | --- |
| PeNGaRoo | **0.8235** | 0.7353 | 0.7568 | 0.7794 | 0.5610 | 0.7887 | 0.8521 | 0.9042 |
| ASPIRER | **0.8235** | **0.9412** | **0.9333** | **0.8824** | **0.7701** | **0.8750** | **0.9533** | **0.9444** |

# Table S19. Performance comparison of ASPIRER and NonClasGP-Pred with the similar Recall value.

| Tool | Recall | Specificity | Precision | Accuracy | MCC | F1-score | AUROC | AUPRC |
| --- | --- | --- | --- | --- | --- | --- | --- | --- |
| NonClasGP-Pred | 0.8676 | 0.8529 | 0.8571 | 0.8676 | 0.7356 | 0.8696 | 0.9019 | 0.9177 |
| ASPIRER | **0.8824** | **0.9412** | **0.9375** | **0.9112** | **0.8250** | **0.9091** | **0.9533** | **0.9444** |

# Table S20. Performance comparison of ASPIRER and PeNGaRoo with the same Specificity value.

| Tool | Recall | Specificity | Precision | Accuracy | MCC | F1-score | AUROC | AUPRC |
| --- | --- | --- | --- | --- | --- | --- | --- | --- |
| PeNGaRoo | 0.8235 | **0.7353** | 0.7568 | 0.7794 | 0.5610 | 0.7887 | 0.8521 | 0.9042 |
| ASPIRER | **0.9706** | **0.7353** | **0.7857** | **0.8529** | **0.7263** | **0.8684** | **0.9533** | **0.9444** |

# Table S21. Performance comparison of ASPIRER and NonClasGP-Pred with the same Specificity value.

| Tool | Recall | Specificity | Precision | Accuracy | MCC | F1-score | AUROC | AUPRC |
| --- | --- | --- | --- | --- | --- | --- | --- | --- |
| NonClasGP-Pred | 0.8676 | 0.8529 | 0.8571 | 0.8676 | 0.7356 | 0.8696 | 0.9019 | 0.9177 |
| ASPIRER | **0.9118** | **0.8529** | **0.8611** | **0.8824** | **0.7660** | **0.8857** | **0.9533** | **0.9444** |

# References:

1. Chen Z, Zhao P, Li F et al. iFeature: a python package and web server for features extraction and selection from protein and peptide sequences, Bioinformatics 2018;34:2499-2502.

2. Li F, Leier A, Liu Q et al. Procleave: Predicting Protease-specific Substrate Cleavage Sites by Combining Sequence and Structural Information, Genomics Proteomics Bioinformatics 2020;18:52-64.

3. Song J, Wang Y, Li F et al. iProt-Sub: a comprehensive package for accurately mapping and predicting protease-specific substrates and cleavage sites, Brief Bioinform 2019;20:638-658.

4. Li F, Zhang Y, Purcell AW et al. Positive-unlabelled learning of glycosylation sites in the human proteome, BMC Bioinformatics 2019;20:112.

5. Chen K, Kurgan LA, Ruan J. Prediction of flexible/rigid regions from protein sequences using k-spaced amino acid pairs, BMC structural biology 2007;7:1-13.

6. Usman M, Lee JA. Afp-cksaap: Prediction of antifreeze proteins using composition of k-spaced amino acid pairs with deep neural network. In: 2019 IEEE 19th International Conference on Bioinformatics and Bioengineering (BIBE). 2019, p. 38-43. IEEE.

7. Wang Y, Li F, Bharathwaj M et al. DeepBL: a deep learning-based approach for in silico discovery of beta-lactamases, Brief Bioinform 2020.

8. Li F, Li C, Wang M et al. GlycoMine: a machine learning-based approach for predicting N-, C- and O-linked glycosylation in the human proteome, Bioinformatics 2015;31:1411-1419.

9. Li F, Li C, Revote J et al. GlycoMine(struct): a new bioinformatics tool for highly accurate mapping of the human N-linked and O-linked glycoproteomes by incorporating structural features, Sci Rep 2016;6:34595.

10. Chen Z, Zhao P, Li C et al. iLearnPlus: a comprehensive and automated machine-learning platform for nucleic acid and protein sequence analysis, prediction and visualization, Nucleic acids research 2021;49:e60-e60.

11. Bhasin M, Raghava G. ESLpred: SVM-based method for subcellular localization of eukaryotic proteins using dipeptide composition and PSI-BLAST, Nucleic acids research 2004;32:W414-W419.

12. Saravanan V, Gautham N. Harnessing computational biology for exact linear B-cell epitope prediction: a novel amino acid composition-based feature descriptor, Omics: a journal of integrative biology 2015;19:648-658.

13. Govindan G, Nair AS. Composition, Transition and Distribution (CTD)—a dynamic feature for predictions based on hierarchical structure of cellular sorting. In: 2011 Annual IEEE India Conference. 2011, p. 1-6. Ieee.

14. Tomii K, Kanehisa M. Analysis of amino acid indices and mutation matrices for sequence comparison and structure prediction of proteins, Protein Engineering, Design and Selection 1996;9:27-36.

15. Chou KC. Prediction of protein cellular attributes using pseudo‐amino acid composition, Proteins: Structure, Function, and Bioinformatics 2001;43:246-255.

16. Chou K-C, Shen H-B. MemType-2L: a web server for predicting membrane proteins and their types by incorporating evolution information through Pse-PSSM, Biochemical and biophysical research communications 2007;360:339-345.

17. Kawashima S, Pokarowski P, Pokarowska M et al. AAindex: amino acid index database, progress report 2008, Nucleic acids research 2007;36:D202-D205.

18. Feng Z-P, Zhang C-T. Prediction of membrane protein types based on the hydrophobic index of amino acids, Journal of protein chemistry 2000;19:269-275.

19. Yadav S, Shukla S. Analysis of k-fold cross-validation over hold-out validation on colossal datasets for quality classification. In: 2016 IEEE 6th International conference on advanced computing (IACC). 2016, p. 78-83. IEEE.

20. Stone M. Cross-validation: A review, Statistics: A Journal of Theoretical and Applied Statistics 1978;9:127-139.

21. Zhao L, Chen J, Sun J et al. Multimer recognition and secretion by the non-classical secretion pathway in Bacillus subtilis, Scientific reports 2017;7:1-18.

22. Vacic V, Iakoucheva LM, Radivojac P. Two Sample Logo: a graphical representation of the differences between two sets of sequence alignments, Bioinformatics 2006;22:1536-1537.

23. Kang Q, Zhang D. Principle and potential applications of the non-classical protein secretory pathway in bacteria, Applied microbiology and biotechnology 2020;104:953-965.
